# Efficacy of Lateral Sagittal Versus Costoclavicular Approach in Ultrasound-Guided Infraclavicular Block for Below Elbow Surgery: A Randomized Controlled Trial

**DOI:** 10.7759/cureus.107586

**Published:** 2026-04-23

**Authors:** Jishmi K Shah, Vaibhavi Hazariwala, Birva Khara, Bhumika K Patel

**Affiliations:** 1 Anesthesiology, Pramukhswami Medical College, Bhaikaka University, Karamsad, IND; 2 Anesthesiology, Dr. Kiran C. Patel Medical College and Research Institute, Bharuch, IND

**Keywords:** below elbow surgeries, infraclavicular brachial plexus block, patient’s satisfaction, surgeon satisfaction, ultrasound guided regional anaesthesia, usg guided brachial plexus block

## Abstract

Background

The infraclavicular brachial plexus block is a widely used regional anesthesia technique for below-elbow surgeries. The lateral sagittal (LS) and costoclavicular (CC) approaches differ anatomically and technically, potentially affecting block performance and outcomes. This study aimed to compare the efficacy of the LS and CC approaches for ultrasound-guided infraclavicular brachial plexus block in terms of block success, procedural time, onset of sensory and motor blockade, and patient and surgeon satisfaction.

Methods

A randomized controlled trial was conducted between April 2024 to April 2025 at Shree Krishna Hospital, Karamsad, Gujarat, a tertiary care teaching hospital involving 48 adult patients undergoing elective below-elbow surgery, randomized equally to receive 22 mL of local anesthetic solution (20 mL of 0.5% ropivacaine mixed with 2 mL of 8 mg of dexamethasone) either by the LS or CC approach. Block performance time, sensory and motor block onset, satisfaction scores, and complications were assessed.

Results

The CC approach demonstrated a significantly shorter block performance time [median: 8 minutes (IQR 6-8)] compared to the LS approach [median: 10 minutes (IQR 8-12.3)] (p < 0.001), with a median difference of 2.05 minutes (95% CI: -5.5 to 9.0). The onset of sensory blockade was shorter in the CC group [15 minutes (IQR 15-20)] compared to the LS group [20 minutes (IQR 15-20)], although this difference was not statistically significant (p = 0.145; median difference 0.0 minutes, 95% CI: -5 to 10). A statistically significant difference was observed in the onset of motor blockade (p = 0.012); however, median values were similar between groups, with a median difference of 0.0 minutes (95% CI: -5 to 10), suggesting limited clinical relevance. Patient and surgeon satisfaction were high and comparable between groups (odds ratio: 0.64, 95% CI: 0.10-4.20), and no complications were reported.

Conclusion

The costoclavicular approach is associated with shorter block performance time, while demonstrating comparable sensory and motor blockade characteristics and satisfaction outcomes when compared to the lateral sagittal approach. These findings suggest that both techniques are effective and clinically acceptable options for infraclavicular brachial plexus block in below-elbow surgeries. Further large-scale, multicenter studies are warranted to confirm these findings and to evaluate additional clinically relevant outcomes, including duration of analgesia and operator learning curves.

## Introduction

Regional anesthesia has become an integral component of modern perioperative care, particularly for upper limb surgeries, where it provides superior analgesia, reduces opioid consumption, and facilitates early recovery. Among various regional techniques, the infraclavicular brachial plexus block is widely utilized for surgeries involving the forearm, wrist, and hand, owing to its reliability in providing dense sensory and motor blockade distal to the elbow [1]. The advent of ultrasound guidance has further enhanced the safety and efficacy of this block by enabling the immediate visualization of neural structures and adjacent vessels, and needle trajectory, thereby minimizing complications and improving block success rates [2,3].

The infraclavicular block targets the cords of the brachial plexus, which are anatomically arranged around the axillary artery at the level below the clavicle [4]. Traditionally, several approaches have been described, including the classic infraclavicular approach, vertical infraclavicular block, and more recently, ultrasound-guided techniques such as the lateral sagittal (LS) and costoclavicular (CC) approaches [3,5]. Each of these approaches differs in terms of anatomical landmarks, needle insertion point, spread of local anesthetic, and potential for achieving complete blockade.

The lateral sagittal approach, one of the earlier ultrasound-guided techniques, involves placing the transducer in a parasagittal orientation below the clavicle to visualize the axillary artery and surrounding cords; however, its effectiveness may be limited by the deeper location and variable anatomy of the cords, leading to potential incomplete blockade, especially of the posterior cord, and suboptimal needle visualization in some patients [4,6,7]. In contrast, the costoclavicular approach, a relatively newer technique, positions the transducer parallel and inferior to the clavicle, enabling visualization of all three cords in a more superficial, compact arrangement lateral to the axillary artery, which facilitates easier needle guidance, better local anesthetic spread, and potentially higher success rates with improved block consistency and reduced anesthetic volume [4,8-11].

Achieving an effective brachial plexus block requires both adequate sensory blockade for analgesia and sufficient motor blockade for optimal surgical conditions. While several studies have compared different infraclavicular approaches, variability exists in reported outcomes regarding onset time, completeness of blockade, procedural ease, and complication rates [8-10,12]. The definition of block success varies across studies, with some assessing only sensory blockade while others include both sensory and motor components; however, inadequate motor blockade may compromise surgical conditions and necessitate conversion to general anesthesia, highlighting the need for comprehensive evaluation. While interest in the costoclavicular approach is growing, there is little evidence comparing it to the lateral sagittal approach for combined sensory and motor block success in below-elbow surgeries. So, this study aimed to compare these two ultrasound-guided infraclavicular approaches in terms of block success while also evaluating procedural time and patients' and surgeons’ satisfaction. We hypothesized that the CC approach would result in favorable block characteristics compared to the LS approach.

## Materials and methods

Study design, duration, and setting

This prospective, parallel-group randomized controlled trial was conducted between April 2024 and April 2025 at Shree Krishna Hospital, Karamsad, Gujarat, a tertiary care teaching hospital associated with Pramukhswami Medical College, affiliated with Bhaikaka University, Karamsad, Anand, Gujarat.

Study population

Patients scheduled for elective below-elbow surgeries under infraclavicular brachial plexus block were enrolled in the study.

Inclusion criteria

Adult patients aged 18-65 years, classified as American Society of Anesthesiologists (ASA) physical status I-III [13], scheduled for elective below-elbow surgery under infraclavicular brachial plexus block, and providing written informed consent were included in the study.

Exclusion criteria

Patients were excluded if they had a body mass index (BMI) greater than 30 kg/m², contraindications to regional anesthesia, infection at the infraclavicular fossa or refusal to participate. Additional exclusion criteria included a history of hypersensitivity or allergy to local anesthetic agents, presence of severe renal, cardiac, or hepatic disease, bleeding disorders or coagulopathy, pre-existing neurological deficits, psychiatric illness, and pregnancy. Patients in whom the block was inadequate and required conversion to general anesthesia were also excluded from the final analysis.

Sample size

The sample size was calculated using OpenEpi (Version 3) for comparison of two independent means. A two-sided confidence level of 95% and a study power of 80% were considered, with an equal allocation ratio between the two groups (1:1). Based on data from a prior study, the standard deviations of block performance time for the lateral sagittal (LS) approach was 1.74 minutes and 1.94 minutes for the costoclavicular (CC) approach, with an expected mean difference of 1.5 minutes between the groups [14]. Using these parameters, the calculated sample size was 24 patients per group. Accordingly, the study comprised a total of 48 participants, with each group consisting of 24 individuals.

Randomization and allocation concealment

**Figure 1:**
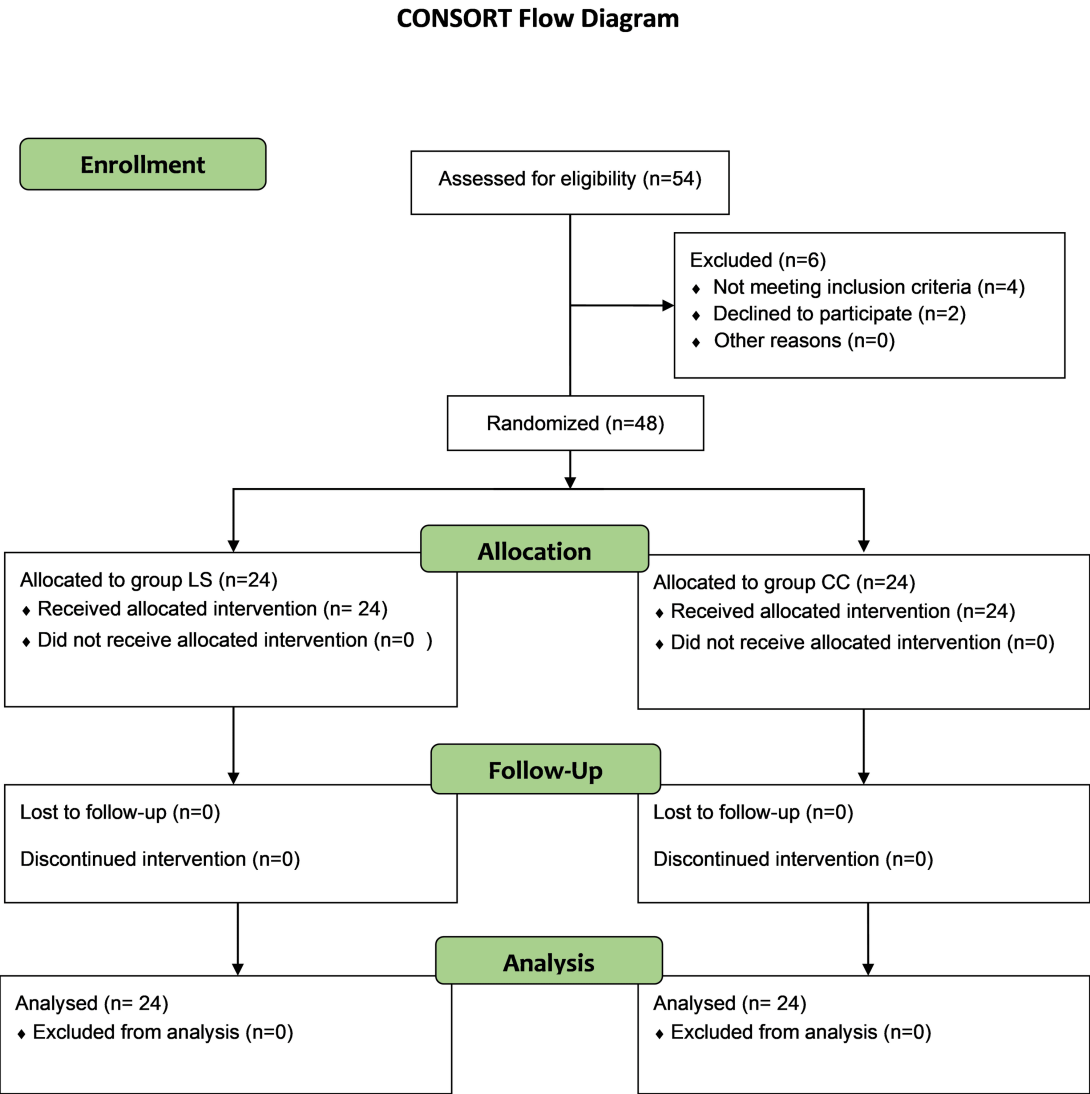
CONSORT flow diagram LS: Lateral sagittal; CC: costoclavicular

Participants were randomly assigned to two groups (Group LS: lateral sagittal approach; Group CC: costoclavicular approach) of equal size (n=24 each) utilizing a computer-generated randomization sequence. Allocation concealment was ensured using sequentially numbered, sealed opaque envelopes, which were opened immediately prior to the procedure. The randomization sequence was generated by an independent investigator who was not involved in patient enrollment or outcome assessment. Figure 1 illustrates the participant enrollment and randomization process, following the CONSORT guidelines.

Blinding

Due to the nature of the intervention, the anesthesiologist performing the block was not blinded. However, participants, surgeons performing surgery, outcome assessor evaluating sensory and motor blockade, and the statistician analyzing the data were blinded to group allocation.

Interventions

All patients underwent a pre-anesthetic evaluation, including a detailed clinical history and comprehensive general and systemic examination. Intravenous access was secured prior to the procedure. All patients were monitored using standard monitoring (electrocardiogram, non-invasive blood pressure, and pulse oximetry). An ultrasound-guided infraclavicular brachial plexus block was performed under strict aseptic precautions using an 8-12 MHz linear probe with color doppler. All blocks were performed by an experienced anesthesiologist proficient in ultrasound-guided regional anesthesia.

Local Anesthetic Solution

In both groups, a total of 22 mL of local anesthetic solution was given, consisting of 20 mL of 0.5% ropivacaine mixed with 2 mL containing 8 mg of dexamethasone.

Group LS (Lateral Sagittal Approach)

In patients allocated to the LS group, the ultrasound probe was placed medial to the coracoid process in a parasagittal plane in the infraclavicular region to visualize the three cords of the brachial plexus around the axillary artery. A 23-gauge spinal needle was advanced in-plane in a cephalocaudal direction toward the cords, and following negative aspiration, the local anesthetic solution was deposited incrementally around the posterior (8 mL), lateral (8 mL), and medial (6 mL) cords.

Group CC (Costoclavicular Approach)

In the CC group, the ultrasound probe was placed parallel and inferior to the clavicle to visualize the costoclavicular space. The three cords of the brachial plexus were identified lateral to the axillary artery in a clustered arrangement. The needle was advanced in-plane toward the center of the cords, and following negative aspiration, the local anesthetic solution was injected to achieve uniform spread around all three cords.

Outcome measures

The primary outcome of the study was the block success rate at 30 minutes, defined as the achievement of complete sensory and motor blockade within 30 minutes of local anesthetic administration. Secondary outcomes included block performance time, onset time of sensory blockade, onset time of motor blockade, incidence of complications or adverse events, and patient and surgeon satisfaction.

The block performance time was defined as the time from the insertion of the needle into the skin until the block needle was removed from the skin after the completion of the procedure. Sensory block was assessed using the pinprick method in the distribution of the median, ulnar, radial, and musculocutaneous nerves. The degree of sensory blockade for each nerve was graded using a three-point scale adapted from Koscielniak-Nielsen: score 2 = complete sensory block (absence of both touch and pain sensation), score 1 = partial sensory block (presence of touch sensation but absence of pain), and score 0 = no block (sharp pain sensation present) [15]. Quality of motor block was assessed using a four-point scale adapted from Abhinaya et al.: score 3 = complete motor block (no movement in the entire upper limb), score 2 = partial motor block (movement present in the hand but not in the arm), score 1 = reduced motor strength (both hand and arm movements possible against gravity but not against resistance), and score 0 = no motor block (full movement in both hand and arm against resistance) [16]. The onset of sensory block was defined as the time elapsed between injection of the drug and complete loss of pinprick sensation, whereas the onset of motor blockade was defined as the time interval from injection of the local anesthetic to the achievement of complete motor blockade. Patient and surgeon satisfaction were evaluated using a single-item, five-point Likert-type item ranging from 1 (very dissatisfied) to 5 (very satisfied).

The onset and degree of sensory and motor block were evaluated at 5-minute intervals for up to 30 minutes. Failure to achieve complete sensory and motor blockade within 30 minutes was considered a failure of the block.

Data collection

Following the procedure, an independent anesthesiologist, blinded to the technique used, assessed both sensory and motor blockade. The onset and degree of sensory and motor block were evaluated at 5-minute intervals for up to 30 minutes or until complete blockade was achieved. Patient and surgeon satisfaction, as well as the occurrence of any adverse events, were also recorded in both groups.

Failure of block, defined as inadequate sensory and motor blockade necessitating conversion to general anesthesia, was noted and such cases were excluded from the final analysis. Demographic variables, block characteristics, and outcome measures were documented using a standardized data collection proforma by an independent observer blinded to group allocation.

Statistical analysis

Statistical analysis was performed using IBM Statistical Package for the Social Sciences (SPSS) for Windows, Version 26 (Released 2018; IBM Corp., Armonk, NY, USA). Normality assumptions were checked for all the variables by the Kolmogorov-Smirnov test. Quantitative variables were expressed as mean ± standard deviation (SD) or median along with interquartile range (IQR), as appropriate. Qualitative variables were expressed as frequencies and percentages. Satisfaction scores were categorized into three groups for analysis: scores of 1 and 2 (very dissatisfied and dissatisfied) were classified as “dissatisfied,” scores of 4 and 5 (satisfied and very satisfied) as “satisfied,” and score 3 was retained as “neither satisfied nor dissatisfied.” These categories were subsequently analyzed as categorical data derived from a single-item Likert-type measure. The Mann-Whitney U test was used to compare continuous variables between the two groups, and between-group differences were expressed as median differences with 95% confidence intervals. Categorical variables were compared using the Chi-square test or Fisher’s exact test, as appropriate. For significant or clinically relevant categorical outcomes, effect sizes were reported as odds ratios with 95% confidence intervals. A p-value of less than 0.05 was considered statistically significant.

Ethical issues

The study protocol was approved by the Institutional Ethics Committee (IEC/BU/151/Faculty/12/96/2024), and registered in the Clinical Trials Registry - India (CTRI) (CTRI/2025/01/079412). Written informed consent was obtained from all participants. Patient confidentiality was maintained throughout the study. Management of cases was done according to standard guidelines.

## Results

The mean age of patients was 44.2 (± 14.4) years in the LS group and 46.1 (± 18.2) years in the CC group. The gender distribution was identical in both groups, with 16 males (66.7%) and 8 females (33.3%) in each group. Most patients in both groups belonged to ASA class II. Overweight was the most common category in the LS group (11/24, 45.8%), whereas obesity class I was most common in the CC group (15/24, 62.5%). Regarding comorbidities, 14 patients (58.3%) in the LS group had associated comorbid conditions compared with 10 patients (41.7%) in the CC group. Detailed baseline characteristics are depicted in Table 1.

**Table 1:** Baseline characteristics of the participants (n=48) LS: Lateral sagittal; CC: costoclavicular; ASA: American Society of Anesthesiologists; BMI: body mass index

Variables	Group LS (n=24)	Group CC (n=24)
Age (in years) (mean ± SD)	44.2 ± 14.4	46.1 ± 18.2
Gender		
Male	16 (66.7%)	16 (66.7%)
Female	8 (33.3%)	8 (33.3%)
ASA Classification		
I	1 (4.2%)	0 (0.0%)
II	18 (75.0%)	18 (75.0%)
III	5 (20.8%)	6 (25.0%)
BMI		
Normal (18.5-22.9 kg/m^2^)	7 (29.2%)	2 (8.3%)
Overweight (23-24.9 kg/m^2^)	11 (45.8%)	7 (29.2%)
Obesity class I (25-29.9 kg/m^2^)	6 (25.0%)	15 (62.5%)
Comorbidities		
Present	14 (58.3%)	10 (41.7%)
Absent	10 (41.7%)	14 (58.3%)

The comparison of block characteristics between the LS approach and the CC approach is presented in Table 2. The CC approach demonstrated a significantly shorter block performance time compared to the LS approach. The median block performance time was significantly lower in the CC group [8 minutes (IQR 6-8)] compared with the LS group [10 minutes (IQR 8-12.3)] (Mann-Whitney U=114, Z=-3.673, p<0.001), with a Hodges-Lehmann median difference of 2.05 minutes (95% CI: -5.5 to 9.0). The CC group demonstrated a shorter median onset time of sensory blockade [15 minutes (IQR: 15-20)] compared with the LS group [20 minutes (IQR: 15-20)]. However, this difference did not reach statistical significance (Mann-Whitney U=225, Z=-1.458, p=0.145), with a median difference of 0.0 minutes (95% CI: -5 to 10). In contrast, the onset of motor blockade differed significantly (Mann-Whitney U=193.5, Z=-2.501, p=0.012) between the groups. The LS group showed a median onset of 25 minutes (IQR: 25-25), whereas the CC group had a median onset of 25 minutes (IQR: 20-25), with a median difference of 0.0 minutes (95% CI: -5 to 10).

**Table 2:** Comparison of block characteristics LS: Lateral sagittal; CC: costoclavicular; IQR: interquartile range; HL: Hodges–Lehmann; CI: confidence interval; *Statistically significant

Variable	Group LS (n=24)	Group CC (n=24)	Mann-Whitney U	Z	p-value	Median Difference (HL, 95% CI)
	Median (IQR)	Median (IQR)				
Block performance time (in minutes)	10 (8–12.3)	8 (6–8)	114	-3.673	<0.001*	2.05 (-5.5 to 9.0)
Onset of sensory blockade (in minutes)	20 (15–20)	15 (15–20)	225	-1.458	0.145	0.0 (-5 to 10)
Onset of motor blockade (in minutes)	25 (25–25)	25 (20–25)	193.5	-2.501	0.012*	0.0 (-5 to 10)

Patients’ and surgeons’ satisfaction following the procedure was particularly high in both cohorts (Table 3). In the LS cohort, 22 (91.7%) patients expressed satisfaction, whereas two (8.3%) patients were neither satisfied nor dissatisfied. Similarly, in the CC cohort, 21 (87.5%) patients reported satisfaction, and three (12.5%) patients indicated a neutral stance. A comparative analysis using Fisher’s exact test revealed no statistically significant difference in patient satisfaction between the LS and CC approaches (p=1.000), with an odds ratio of 0.64 (95% CI: 0.10-4.20). Surgeon satisfaction was also high across both study cohorts (Table 3). In the LS group, 22 (91.7%) surgeons reported satisfaction, while two (8.3%) were neither satisfied nor dissatisfied. In the CC group, 21 (87.5%) surgeons were satisfied, and three (12.5%) reported neutral satisfaction. Statistical evaluation using Fisher’s exact test demonstrated no significant difference in surgeon satisfaction between the two cohorts (p=1.000), with an odds ratio of 0.64 (95% CI: 0.10-4.20). There was no complication reported in either of the groups.

**Table 3:** Comparison of patient’s and surgeon’s satisfactions between groups (n=48) LS: Lateral sagittal; CC: costoclavicular; Values expressed as n (%) within each group; ^#^p-value calculated using Fisher’s exact test; CI: confidence interval

Outcome	Group	Neither satisfied/dissatisfied	Satisfied	p-value^#^	Odds Ratio (95% CI)
Patient satisfaction	LS	2 (8.3)	22 (91.7)	1.000	0.64 (0.10-4.20)
	CC	3 (12.5)	21 (87.5)		
Surgeon satisfaction	LS	2 (8.3)	22 (91.7)	1.000	0.64 (0.10-4.20)
	CC	3 (12.5)	21 (87.5)		

## Discussion

The present randomized study evaluated the effectiveness of the LS and CC approaches for ultrasound-guided infraclavicular brachial plexus block in below-elbow surgeries, with particular emphasis on block performance time, onset of sensory and motor blockade, and satisfaction outcomes. The findings demonstrate that the CC approach offers a significantly shorter block performance time and faster onset of motor blockade, while both techniques provide comparable sensory onset and high satisfaction rates.

The median block performance time in this study for CC approach was 8 minutes (IQR:6-8), while for LS approach was 10 minutes (IQR: 8-12.3). This demonstrated a clear procedural advantage of the CC technique, with a statistically significant reduction in performance time. This finding aligns well with existing literature that emphasizes the procedural efficiency of the CC approach. Previous studies by Dost et al., Yayik et al. and Li et al. had similarly reported shorter block performance times with the CC technique [9,12,17]. These authors attributed the improved efficiency primarily to superior sonographic visualization of the cords clustered lateral to the axillary artery in the costoclavicular space, which facilitates quicker identification of anatomical landmarks. In addition, the requirement for fewer needle redirections due to the compact arrangement of neural structures contributes to a smoother and faster procedure. The feasibility of achieving an effective block with a single injection in the CC approach further enhances its time efficiency, reducing both procedural complexity and operator dependency [9,12,17,18]. In contrast, Kaya et al. reported a shorter block performance time with the LS approach compared to the CC approach; however, this difference did not reach statistical significance [10]. Furthermore, Cesur et al. also demonstrated that the CC approach offers improved procedural efficiency, particularly in patients with difficult or variable anatomy, where conventional approaches may be more challenging and time-consuming [8]. The findings of the present study corroborate these observations, reinforcing the procedural advantage of the CC technique. This reduction in block performance time is clinically relevant, especially in high-throughput surgical settings where efficiency, turnover time, and resource optimization are critical. Collectively, these results suggest that the CC approach not only simplifies the technical aspects of infraclavicular block but also enhances overall procedural workflow without compromising effectiveness. Though there was statistical significance, the Hodges-Lehmann median difference with 95% confidence interval showed a difference of 2.05 minutes (95% CI: -5.5 to 9.0), indicating variability in the estimate and suggesting that the magnitude of benefit, although statistically significant, should be interpreted with caution in terms of clinical significance.

In the present study, the onset of sensory blockade was comparable between groups, with a shorter median onset observed in the CC group [15 minutes (IQR: 15-20)] compared with the LS group [20 minutes (IQR: 15-20)], although this difference did not reach statistical significance. The median difference with 95% confidence interval [0.0 minutes (95% CI: -5 to 10)] further supports the absence of a meaningful difference between the two techniques. A similar trend toward faster sensory onset with the CC approach has been reported in previous studies; however, unlike our findings, Cesur et al. and Dost et al. demonstrated statistically significant differences [8,9]. Dost et al. reported median sensory onset times of 5 minutes (IQR: 4.49-6; 95% CI: 5-6) in one group and 4 minutes (IQR: 3.88-6; 95% CI: 4-5) in the other, indicating a faster onset with the CC approach [9]. Similarly, Cesur et al. observed a significantly shorter sensorimotor onset time in the CC group [(15.95 ± 2.97) minutes] compared with the LS group [(17.72 ± 4.15) minutes], suggesting a potential advantage of the CC technique that may not have been evident in the present sample [8]. This finding may be explained by the fact that both approaches ultimately target the same neural structures - the cords of the brachial plexus - while the pharmacodynamic properties of the local anesthetic remain constant. Consequently, although the anatomical approach may influence technical ease and needle handling, it is unlikely to substantially affect sensory onset when adequate drug distribution is achieved. However, under conditions of reduced local anesthetic volume, anatomical factors may assume greater importance. In this context, Tran et al. reported a modestly faster onset with the CC approach, suggesting that the compact arrangement of cords in this region may facilitate more efficient drug spread when volume is limited [19]. The present study demonstrated a statistically significant difference in the onset of motor blockade in the CC group compared to the LS group. However, the median values were similar, and the Hodges-Lehmann estimate [0.0 minutes (95% CI: -5 to 10)] indicates a minimal absolute difference, suggesting that the observed statistical significance may not translate into a clinically meaningful advantage. This finding is consistent with previous studies that have similarly demonstrated a faster and more reliable motor blockade with the costoclavicular approach [8,9,12,18]. A plausible explanation lies in the anatomical configuration of the costoclavicular space, where the cords are closely clustered, allowing more uniform spread of local anesthetic - particularly around the posterior cord, which is frequently associated with incomplete motor blockade in the LS approach [19]. In contrast, the relatively deeper location and greater anatomical variability encountered with the LS approach may contribute to delayed or inconsistent motor blockade, as suggested by previous anatomical and clinical studies.

Another important observation from this study was the comparable patient and surgeon satisfaction between the two groups. Both approaches demonstrated high satisfaction rates, with no statistically significant difference. The odds ratio analysis [OR: 0.64 (95% CI: 0.10-4.20)] further confirms the absence of a meaningful difference, although the wide confidence interval reflects limited precision due to the small sample size. This indicates that despite differences in procedural and block characteristics, both techniques are clinically acceptable and effective. Previous studies have also reported similar satisfaction outcomes, suggesting that as long as adequate anesthesia is achieved, patient-perceived outcomes remain favorable regardless of the approach used [9,10,14,18]. It is noteworthy that satisfaction is a multifactorial outcome influenced not only by block success but also by perioperative comfort, absence of complications, and overall surgical experience. The findings of this study have practical implications. The shorter performance time and faster motor onset associated with the CC approach may improve operating room efficiency and workflow, particularly in high-volume settings. Additionally, the improved needle visualization and superficial location of the cords in the CC approach may enhance safety, especially for less experienced practitioners. However, it is important to note that both techniques require proficiency in ultrasound-guided regional anesthesia, and operator experience remains a critical determinant of success. No complications were observed in either group in the present study. However, existing evidence indicates potential safety advantages of the CC approach, including a reduced risk of pneumothorax due to more lateral probe placement, improved visualization of vascular structures, and a lower likelihood of inadvertent vascular puncture. The relatively superficial position of the cords within the costoclavicular space may also facilitate improved needle control, thereby enhancing procedural safety, particularly for less experienced operators [11].

Limitations

While the relatively small sample size may limit the clinical significance and generalizability of the findings, longer-term outcomes, including duration of analgesia, were not assessed, restricting a more comprehensive evaluation of clinical efficacy. In addition, satisfaction was assessed using a single-item Likert scale, which may not fully capture the multidimensional aspects of patient and surgeon satisfaction. Although no cases of block failure or loss to follow-up were encountered in the present study, and all randomized participants were included in the final analysis, the absence of a formal intention-to-treat analysis may still be considered a methodological limitation. In studies of regional anesthesia, exclusion of failed blocks can introduce selection bias and potentially overestimate the true effectiveness of a technique.

## Conclusions

This study suggests that the CC approach to infraclavicular brachial plexus block is associated with shorter block performance time, while demonstrating comparable sensory onset, motor blockade characteristics, and satisfaction levels of patients and surgeons when compared with the LS approach. These findings indicate that both techniques are effective and clinically acceptable options for below-elbow surgeries. Further research through larger, multicenter trials is warranted to confirm these results and to evaluate additional clinically relevant outcomes, including duration of analgesia and operator learning curves. Studies examining varying volumes of local anesthetic within the CC approach may also help refine dosing strategies in this anatomical region.
